# Pulse blood pressure and cardiovascular mortality in a population-based cohort of elderly Costa Ricans

**DOI:** 10.1038/jhh.2015.117

**Published:** 2015-12-17

**Authors:** L Rosero-Bixby, F Coto-Yglesias, W H Dow

**Affiliations:** 1 Centro Centroamericano de Población (CCP), Universidad de Costa Rica, San Pedro, Costa Rica; 2 School of Public Health, the University of California, Berkeley, CA, USA; 3 Servicio de Geriatría, Hospital Nacional de Geriatría y Gerontología, San José, Costa Rica

## Abstract

We studied the relationships between blood pressure (BP), pulse pressure (PP) and cardiovascular (CV) death in older adults using data from 2346 participants enrolled in the Costa Rican CRELES study, mean age 76 years (s.d. 10.2), 31% qualified as wide PP. All covariates included and analyzed were collected prospectively as part of a 4-year home-based follow-up; mortality was tracked for an additional 3 years, identifying 266 CV deaths. Longitudinal data revealed little change over time in systolic BP (SBP), a decline in diastolic BP, and widening of PP. Wide PP was associated with higher risk of CV death but only among individuals receiving antihypertensive drug therapy. Individuals with both wide PP and receiving therapy had 2.6 hazard rate of CV death relative to people with normal-PP plus not taking treatment (TRT), even adjusting for SBP. Increasing PP between visits was significantly associated to higher CV death independently of TRT status. SBP and DBP were not significantly associated to CV death when the effect of PP was controlled for. Conclusion: elderly hypertensive patients with wide or increasing PP, especially if receiving TRT, are the highest CV risk group, thus must be carefully assessed, monitored and treated with caution.

## Introduction

Cardiovascular (CV) diseases are the main cause of morbidity and mortality globally with hypertension being responsible for about half of deaths due to heart disease and stroke.^1^ Given that blood pressure (BP) in modern populations increases with age, especially in mid-adult ages, prevalence of high BP among elderly people is very high:^2^ about 70% of Latin Americans aged 70 years or more are hypertensive;^3^ similarly, only 7% of individuals aged 80 or more had normal BP in the Framingham study.^4^ Prevalence of hypertension is higher in low- and middle-income countries than in the more developed countries^5^ and, because of population aging and behavioral factors such as smoking, salt intake, obesity or reduced physical activity, prevalence of hypertension is increasing globally.^6^ In turn, the nil prevalence of hypertension, and of its increase with age, observed in indigenous populations^7^ suggests that environmental conditions is the primary origin of this modern-world disease.

Although the role of high BP on CV health among the elderly has been well documented in high-income populations, there is scarce data on the prevalence, management and relative importance of different BP indices on CV risk among elderly from low- and middle-income countries. Several studies suggest that the association between BP and adverse CV events differs across elderly populations from different countries,^8, 9, 10^ but the mechanisms are not well understood. In lower income countries, higher mortality due to competing causes, such as infectious diseases and renal diseases, as well as differences in the prevalence of other CV risk factors could reduce or strengthen the relationship between BP and CV mortality.^11, 12^

Of particular interest in this study is the relative role of systolic BP (SBP), diastolic BP (DBP) and pulse pressure (PP) in predicting older adult mortality in a developing country context. PP is the difference between SBP and DBP, and has been suggested as an important independent CV risk factor.^2, 13, 14^ Epidemiological studies have shown that among middle-aged people, SBP and DBP rise with age in a parallel manner and thus PP is constant over time, but after about age 50 years, only SBP continues to rise and thus a widening PP emerges as a relevant vital sign among older individuals.^15^

The general objective of this article was to identify the association of high BP with CV death in a cohort of elderly Costa Ricans from the population-based panel named ‘Costa Rican Longevity and Healthy Aging Study' or CRELES.^16^ A first specific objective was to test the hypothesis that wide PP is an important component of high BP associated with increased risk of CV death in the elderly independently of SBP and DBP. Our second specific objective was to assess the association of changes over time in the components of BP with CV mortality. And third, transversal, specific objective was to assess the modificating effect of antihypertensive drug therapy on the association BP-mortality, as background for future work to refine treatment (TRT) regimens.

The Costa Rican data provide a rare opportunity in that they allow a long-term longitudinal mortality follow-up in a developing country population. Costa Rica is a small middle-income country with a rapidly aging population and a remarkably high life expectancy of 79 years in 2011.^17^ Its public health care and insurance system is near universal, especially for the older population.^18^ Remaining life expectancy of older Costa Rican men is one of the highest in the world due especially to their relatively low CV mortality.^19^ Nevertheless, hypertension prevalence in persons aged 60 years or older is 65% in Costa Rica^20^ compared with 67% in the United States.^21^ About half of hypertensive elderly Costa Ricans have their BP under control, whereas one-fourth are unaware of their condition.^20^ An earlier analysis of CRELES data on the prospective mortality associated to 22 biomarkers showed little to no association between high baseline BP and mortality.^11^ By focusing in a single biomarker in this article we are now able to improve and expand the analysis of the effect of BP and to pursue our three aforementioned specific objectives. We are also able to improve the methodology in several ways, such as reducing regression dilution biases by including multiple longitudinal BP measures over several years in the CRELES panel, instead of just the baseline values used in the earlier study, as well as reducing reverse causation biases by excluding individuals with serious illness at baseline.

## materials and methods

### Study population

We used data from the Costa Rican Study on Longevity and Healthy Aging (CRELES), a longitudinal survey of adults aged 60 or more years and residing in Costa Rica, with oversampling of older ages as described elsewhere.^16^ In brief, CRELES selected a probabilistic, nationally representative sample in a multistage sampling process using the 2000 census as sampling frame. Participants were recruited in 2004–2006, mostly in 2005, in baseline, or wave 1, household visits. The study included two additional waves of home-based interviews and physical exams mostly in 2007 and 2009. All participants signed informed consent and the institutional review board of the University of Costa Rica granted human subjects approval. Sampling weight factors correct oversampling and differences in response rates by age, education and region.

### Outcome: cardiovascular death

Our outcome was death caused by CV diseases. CRELES ascertained mortality and cause of death in two ways: (1) through the computer records in the Costa Rica National Death Registry, and (2) during the second and third waves of home visits. The computer follow-up used the unique identification number (the cédula) that all Costa Ricans have. Surviving individuals were followed in the death registry for 3 years after the last visit. The death registry included >99% of the deaths identified in the longitudinal home visits; by contrast, 10% of the deaths from the Registry were not identified in the fieldwork due to losses to follow-up. For the 3% foreigners in the sample, survival was established only in the field because they do not had a unique identification number with which to link them to the Registry. CV deaths are those with codes I10–I99 (ICD-10) in the main cause of death in the death certificate.

### Exposure variables: BP

Our main exposure variable was wide PP. Trained data collectors took BP in the three CRELES waves with the individual in the sitting position, resting before the measurement, using a cuff located in the right arm and at the heart level, and using the automatic sphygmomanometer OMRON HEM-711AC approved by the European Society of Hypertension International Validation Protocol.^22^ Two measurements 30 min apart from each other were taken during the interview. We used in the analysis the average of these two measures. PP was computed as the difference between the averaged SBP and DBP, and PP⩾70 mm Hg was considered as ‘wide' following Franklin *et al.*^23^

We focused in the association between PP and CV death that is independent of the effects of SBP or DBP. To model SBP we categorized it into three classes: normal (<140 mm Hg), stage-1 SBP hypertension (140–159 mm Hg) and stage-2 SBP hypertension (⩾160 mm Hg). DBP was also categorized into three classes: low (<70 mm Hg), normal (70–89 mm Hg) and high (⩾90 mm Hg).

We assessed the effect modification of antihypertensive therapy using the information about medicines CRELES participants were taking in each visit. The data collectors recorded all the medications shown by participants and study physicians coded medicines by type. Participants taking antihypertensive medicines were considered in antihypertensive therapy, regardless of the purpose of the medication. The majority of patients were taking the Ace-inhibitor enalapril (35%), the calcium channel blocker amlodipine (26%) or the beta blocker atenolol (22%). Diuretics were not counted as antihypertensive. We used TRT, instead of diagnosis, status because of the vagueness of the self reported diagnosis status information available in CRELES – some hypertensive individuals were unaware of their condition and some reportedly hypertensive individuals were not in TRT.

The longitudinal design of CRELES allows to identify changes over time in BP for those who were examined in the second or third waves of visits. We established 5 mm Hg of annual change as the cut-off to identify meaningful increases or decreases in each of the three components (systolic, diastolic and pulse) of BP as continuous variables. For the TRT condition, we defined the transitions between waves from non-TRT to in-TRT and vice versa.

### Other potential confounders

We used the CRELES participants' information on gender, residence in the great metropolitan area of San Jose, years of attained education, self-assessed economic situation in three categories, self-assessed health status as fair or bad, active smoking and body mass index categories for controlling their potential confounding of the relationship between BP and mortality. Participants with baseline reported history of heart attack, stroke or cancer were excluded from the mortality analysis to reduce reverse causation bias.^24^ We used the exact dates of birth, death and visits in the three waves to establish age and survival time. Date of birth, and thus age, did not come from self-report but from the Costa Rican identification card (the *cédula*) validated with the civil registration databases. With the exception of sex and education, all variables, including BP measures, were taken as time-varying covariates with values updated with the information from the most recent interview.

### Statistical analysis

We set the survival-time data with age as the time scale (instead of time as the baseline) as recommended for events like death in which age is more important than time in the study.^25^ The entry point into observation was the date of the baseline visit. The exit point was the date of death or at 3 years after the last visit for survivors. Non-CV deaths were considered as censored observations at the date of death. BP and the other time-varying variables were updated at the date of follow-up visits.

We modeled CV death with parametric proportional hazard models, assuming a Gompertz distribution, which is known to describe well human mortality at old ages.^26^ To model age accurately, we fragmented the time observed for each individual into 1-year-age segments. On average, there were 5.0 segments per individual. We used the STATA MP 12 software(StataCorp LP, College Station, TX, USA) to estimate hazard regression models. All s.e. are robust estimates that correct the lack of independence of the several observations for the same individual. Sampling weights were used in all statistical analyses.

Because PP is a linear combination of SBP and DBP, the three BP components cannot be included in a single model. Therefore, to assess the independent effect of PP we estimated two models: one including PP and SBP and another including PP and DBP.

To show estimates for the general population, all prevalence estimates, and crude CVD-death rates, were computed using all the observations available in CRELES, without the exclusion of seriously ill individuals.

### Sensitivity analysis

To check whether the so called ‘white coat bias' (that is, the first measurement of BP tend to be upward biased^27^) affected our main results we re-estimated them using only the second BP measurement in each visit. To check for detection biases (that is, deaths of hypertensive, and especially in TRT, individuals could be more likely to be classified as CV deaths), we re-estimate our main models for non-CV death as outcome. We also checked the sensitivity of our main results to the use of different cut-offs for wide PP: 65 and 75, instead of 70 mm Hg, as well as to the use of models different than Gompertz regression.

## Results

### Participants and their characteristics

**Figure 1:**
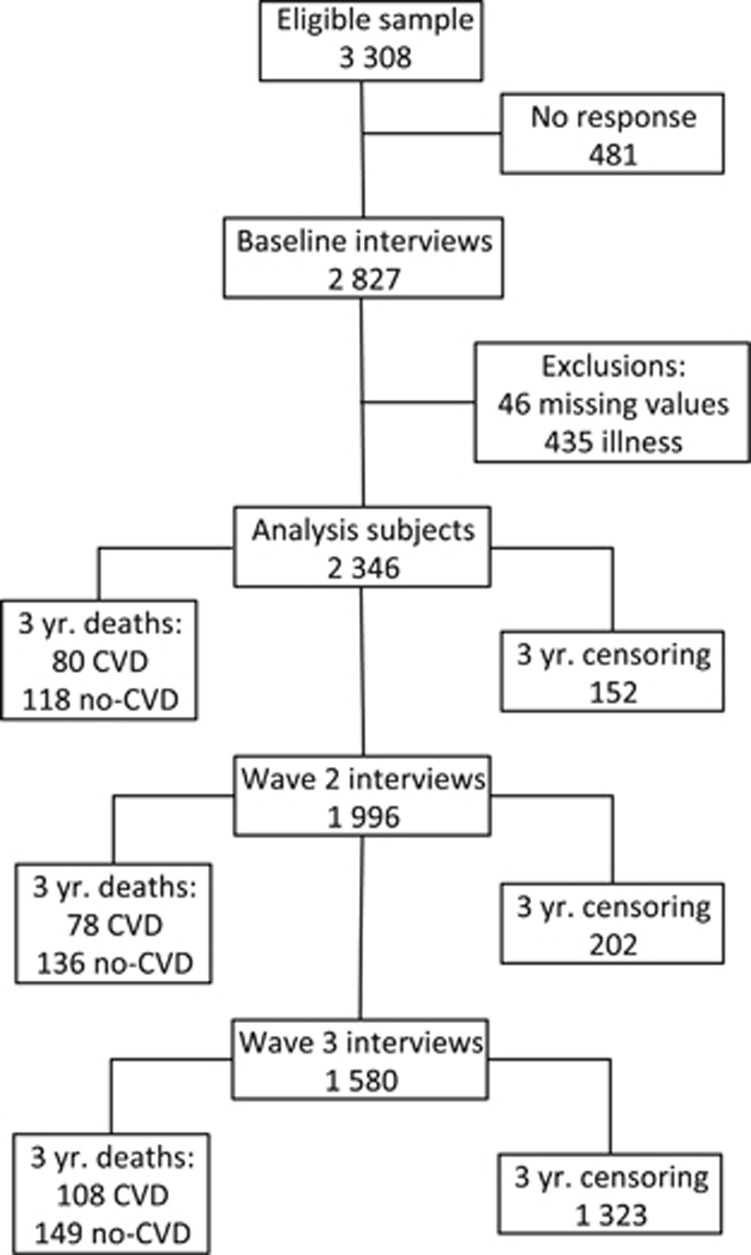
Flowchart of CRELES data used in the main analyses.

The 2827 participants in the original CRELES were 85% of those included in the sample. The bias from higher non-response rates among younger, urban, more affluent individuals was corrected by the sampling weights. In the main analysis of the association of BP and prospective CV mortality, we excluded 46 (2%) individuals with missing variables and 435 (15%) individuals who reported stroke, heart disease or cancer at baseline. The flowchart in Figure 1 shows the numbers of participants and outcomes in the different stages of this longitudinal survey. To estimate the effect of BP levels on mortality our analytical sample size comprised 2346 individuals and 266 CV deaths. To estimate the effect of BP changes, the sample was restricted to individuals with at least two interviews: *N*=1996 and 186 CV deaths. Subjects were visited 2.5 times on average for a mean of 5.1 observation-years.

As shown in Table 1, our main exposure condition—wide PP—included 883 (31%) of participants at baseline (741 after exclusions). About one third of this population was aged 75 years or more after correcting for sampling weights (55% were >75-years-old in the non-weighted sample). The proportion of older individuals was substantially higher in the wide-PP group. Women outnumber men, as usual in an elderly population. About half of this population lived in the great metropolitan area of the capital city. Educational level was low: only 49% had complete elementary school or higher. About 45% of participants self-assessed their economic situation as good or excellent, but 44% reported poor or fair health. Less than 10% were active tobacco smokers and 23% were obese with a body mass index of 30 Kg m^−^^2^ or higher.

### Blood pressure

Almost half (48%) of this population had antihypertensive TRT prescriptions (Table 1); this proportion was higher (60%) in the wide-PP group. More than half of the entire sample meet the criteria for high SBP (SBP⩾140 mm Hg); of these, 31% have SBP indicating stage-1 hypertension (SBP 140–159 mm Hg) and 22% are in stage 2 (SBP⩾160 mm Hg). There was considerable co-occurrence of stage-2 high SBP and wide PP: 62% of wide-PP individuals were in stage-2 high SBP compared with 4% high SBP among the normal-PP group.

Eighteen percent of the entire sample had low DBP (DBP<70 mm Hg). Notably, only 3% of the sample would be considered to have low DBP if the cut-off had been set at 60 mm Hg. Contrary to expectations, low DBP is moderately less common among wide-PP individuals (19 vs 14%). High DBP was a less common contributor to measured hypertension than was high SBP: high DBP affected only 22% of this population (and among those with wide PP, still just 31% had high DBP).

With the 5 mm Hg of annual change as the cut-off to identify meaningful increases or decreases in BP, we found that the same proportion (30%) decreased or increased their SBP between waves (lowest panel in Table 1). In contrast, the predominant change in DBP was decline: 30% compared with 13% with increased DBP. Correspondently with DBP changes, the predominant PP change was increase – 33% compared with 18% with decreasing PP. Although 10% of participants initiated therapy between visits, 4% stopped it. BP changes differed significantly by initial PP status: wide-PP individuals were more likely to reduce their PP and their SBP and to rise their DBP in what looks like a regression to mean change

**Figure 2:**
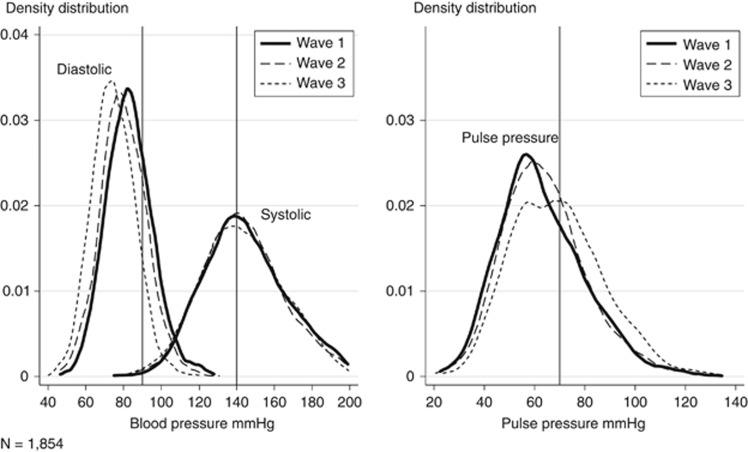
Density distribution of systolic and pulse BP measurements by CRELES wave for participants in all three waves.

Figure 2 shows the sample's distribution (smoothed out with a kernel algorithm implemented in STATA) by the readings in SBP, DBP and PP in each wave of visits. Only individuals with measures in the three waves were represented in the figure; that the three curves are for exactly the same group of individuals and the wave's changes are exclusively due to the passage of time or the ageing of the group (cohort and survival-selection effects are thus controlled). The CRELES' data follows the expected normal-shape distribution in the BP measures. The panel's distribution by SBP did not change across waves. In contrast the DBP distribution shifted to the left with passage of time and the PP distribution shifted to the right. These data thus suggest that aging in this group of elderly Costa Ricans altered little their SBP as a population, whereas it reduced DBP and widened PP.

### BP levels and CV mortality

The crude CV mortality rate in this population was 13 annual deaths per 1000 individuals (Table 2). Among those in TRT or in stage-2 high SBP or with low DBP or with wide-PP the death rate was clearly higher: about 20 per 1000. In the other marginal groups in Table 2 the rate ranges between 7 and 12 per 1000. The lowest rate (seven per 1000) was for individuals who were not in TRT. Stratification by being in TRT revealed that large differences in mortality occured only among people in TRT, in contrast with almost no mortality differences among people not in TRT. For example, crude CV mortality among people in TRT was 32 for those with DBP<70 mm Hg compared with 15 per 1000 among those with high DBP, while the corresponding rates for those not in TRT were 8 and 6, respectively.

The hazard regression estimates simultaneously control the confounding effect of age, sex and some socioeconomic and health characteristics. Table 3 shows the results for BP components from three regression specifications. The first model, which consists of a traditional specification that includes SBP, DBP and TRT, shows that in this elderly population stage-2 high-SBP individuals had 1.89 risk of CV death compared with normal SBP individuals while stage-1 high-SBP individuals did not differ in their CV mortality from those with normal SBP. High DBP seems to reduce the CV hazard to 0.61 (marginally significant effect at *P*=0.06). The risk of individuals taking antihypertensive medicines more than doubled the risk of those not taking medicines.

The second and third model specifications in Table 3 included PP in interaction with the indicator of being in TRT. Adding into the model the effect of PP turned non-significant the associations of DBP or SBP with CV mortality originally observed in the traditional specification. Instead, PP emerged with independent and significant CV mortality effects. The highest CV mortality occured in the group with wide PP and in TRT: 2.82 hazard ratio (HR) relative to the group with normal PP and not in TRT (when DBP was included in the regression), or 2.58 HR when SBP was included in the regression. However, CV mortality in wide-PP individuals did not differ from mortality of those with normal PP among those not taking antihypertensive TRT. If they were in TRT, wide PP was associated with a significant HR of 1.66 compared with normal PP (when DBP was included in the regression), or HR of 1.53 with SBP in the regression.

The possibility of effect modifications by DBP or SBP to the described associations of PP with CV death were not supported by the data. No interaction terms of PP with SBP or DBP categories were statistically significant when added to the models in Table 3 (results not shown).

### BP changes over time and CV mortality

Table 4 shows the estimated effects of changes in BP between visits over prospective CV mortality in addition to BP levels in the starting point of the interval. Only individuals with at least two visits were included, which reduced the number of individuals to 1997. The analysis was stratified by the condition: receiving antihypertensive TRT in the first node of the interval. Only relevant categories of SBP and DBP were modeled as initial levels: stage-2 high SBP (⩾160 mm Hg) and low DBP (<70 mm Hg). Increased PP resulted consistently and significantly associated to higher CV death in the two TRT categories and either in the model with high SBP or in the model with low DBP. The HR of increased PP was 2.6 in the model with SBP and 2.2 in the model with DBP relative to individuals with no change in PP and not in TRT in the initial visit. Starting TRT resulted also significantly associated to higher CV mortality. But the reverse, stopping TRT, was not. Having a wide PP at the beginning of the interval was strongly associated to higher CV death but only in the group receiving therapy, confirming the initial results. SBP and DBP levels and changes were not significantly associated to CV death in these models.

### Sensitivity analyses

Our main results (Table 3) were not sensitive to alternate specifications (Cox regression and Weibull survival distribution models) to our Gompertz regression models (results not shown in tables). Neither, using different cut-offs to define wide-PP altered qualitatively our main results. For example, the HR of 2.58 of wide PP and in TRT individuals in Table 3 was 2.26 for a 65 mm Hg PP cut-off and 2.41 for a 75 cut-off.

Using only the second BP measurement to avoid white coat bias resulted in slightly lower BP readings that reduced the estimated prevalence of high SBP by 1.5 percentage points and that of wide PP by 0.7 points. However, the associations with CV mortality did not changed qualitatively (results shown as Supplementary Information, Supplementary Table SI-2 mirroring Table 3).

Neither individuals receiving antihypertensive TRT nor those with wide-PP showed significantly reduced non-CV mortality, which would had been indication of detection biases in the classification of deaths as of CV origin (results included as Supplementary Information in Supplementary Table SI-2, which mirrors Table 3).

## Discussion

CRELES data for a unique population of elderly individuals from a middle-income country have shown that elderly Costa Ricans with a wide PP (⩾70 mm Hg) and in TRT with antihypertensive medicines were clearly at elevated risk of CV death: HR of 2.6 relative to individuals with normal PP and not in TRT (even after including SBP as a covariate in the regression) and HR 1.5 relative to normal-PP individuals in TRT (HRs were 2.8 and 1.7, respectively, if DBP was included in the regression instead of SBP). Prospectively, individuals with widened PP by 5 mm Hg or more per year showed also a higher CV mortality independently of their TRT status and their initial levels and changes in SBP and DBP. The associations of SBP and DBP with CV death usually disappear when PP is taken into account, especially when recent changes in PP are considered.

In addition to PP, receiving antihypertensive therapy was consistently and significantly associated to higher risk of CV death. Being an observational study, we cannot make causal inferences from this result regarding TRT effectiveness. We cannot distinguish whether clinicians are efficiently targeting the highest risk individuals with TRTs that are lowering risks from even higher levels that they might have experienced in the absence of TRT, vs the alternative hypothesis that TRT regimens are poorly titrated thus have little effect, vs the less likely hypothesis that the TRTs are harmful in this population. An alternative hypothesis is that despite best efforts, TRT regimens were unable to effectively target an underlying cause of increased CV mortality risk, that is, widened PP. Distinguishing between these hypotheses will require future research. However, we can say with confidence that the data show the need to critically examine antihypertensive TRT protocols for older adults in this and similar populations, as well as the need to monitor health and TRT compliance of elderly patients taking antihypertensive medicines who have wide or increasing PP.

In Costa Rica, according to CRELES, about 30% of females and 20% of males in their eighties fall in the group of high risk of CV death, that is, with wide PP and receiving antihypertensive TRT. These proportions double those observed by age 60 years.

DBP of the cohort tends to decline over time and high DBP was not associated with higher risk of CV death. In contrast, SBP stays constant over time in the cohort, which may be in part result of successful antihypertensive TRTs. The fact that BP does not necessarily increase with age, as well as the well-documented absence of hypertension in aboriginal populations, gives us hope for wider prevention efforts. Consistent with other research,^28^ stage-1 high SBP (140–159 mm Hg) does not appear associated with higher CV mortality, which emerges only at stage-2 (SBP⩾160 mm Hg). But even this association disappear when changes over time in PP are taken into account.

Data from the Framingham study shows that DBP is the best CV risk predictor among those younger than age 50, that among age 50–59 a transition stage exists where SBP, DBP and PP are comparable predictors, but after age 60, the DBP is inversely correlated with risk, making the PP a superior predictor than isolated SBP.^14^ Our longitudinal data similarly showed that among older adults, aging had little effect on SBP levels, but it had a clear effect of widening PP. Evidence shows that increase in PP is correlated with heart failure frequency^13^ and global mortality, clarifying discrepancies that are seen at the population level with DBP.^29^

Our data, however, did not support recently reported findings from the Framingham study showing that the higher risk of adverse CV events among elderly patients with wide PP are amplified by (or even limited to individuals with) low DBP (<70 mm Hg) and that CV risk was not greater in people taking antihypertensiv*e*.^30^ DBP levels neither confounded nor modified the higher CV mortality of wide-PP individuals in these CRELES data.

It is well established that the use of antihypertensive drugs in older, stage-2 hyper-SBP patients can be a good intervention to lower CV death risk, but our data raise concerns about the possibility of widening PP and its negative implications on CV mortality. This can be called a ‘hypertension paradox', somewhat similar to the J-curve phenomenon observed in clinical trials and discussed elsewhere.^31, 32, 33^This finding should be weighed against other prevalent comorbid conditions in older adults, such as diabetes, cancer, malnutrition, stroke, dementia, depression and poly-pharmacy that can obscure results in these sub-settings. As we did not analyze specific medications, generalizations must be cautious, but further research is called for.

An evident limitation of this article is that it uses an observational rather than experimental design; thus as discussed above, the results should not be over-interpreted to conclude that the medication has adverse causal effects in some populations (although the results do suggest the importance of further research on that causal question). A causal design is not necessary though to draw our key conclusion: elderly hypertensive patients with wide or increasing PP, especially if receiving TRT, are the highest CV risk group, thus must be carefully assessed, monitored and treated with caution.

A further limitation is inadequate statistical power to detect HRs <2.0, despite the substantial sample size. Given the large effect sizes found though, this did not hinder the main analyses of this paper. However, limited statistical power did preclude disaggregation to more BP categories or their combinations, as well as further stratifying the sample by age groups, sex, frailty or other characteristics.

Among the strengths of this study are its avoidance of regression dilution bias by including several measures of BP and other variables in three waves, its focus on CV mortality (in contrast with other studies of all-cause mortality), its exclusion of individuals with serious diseases, and its population-based character in a nationally representative probabilistic sample that enhances external validity. Providing data from the rarely studied elderly populations outside of high-income countries is also a strength of this article.

In conclusion, our results strengthen the evidence that PP is an independent risk factor of older adult CV mortality. Being an observational study we cannot attribute causality to our results, but we can underscore the need for further experimental studies that assess the effect of TRTs on PP and subsequent mortality at older ages in diverse populations.


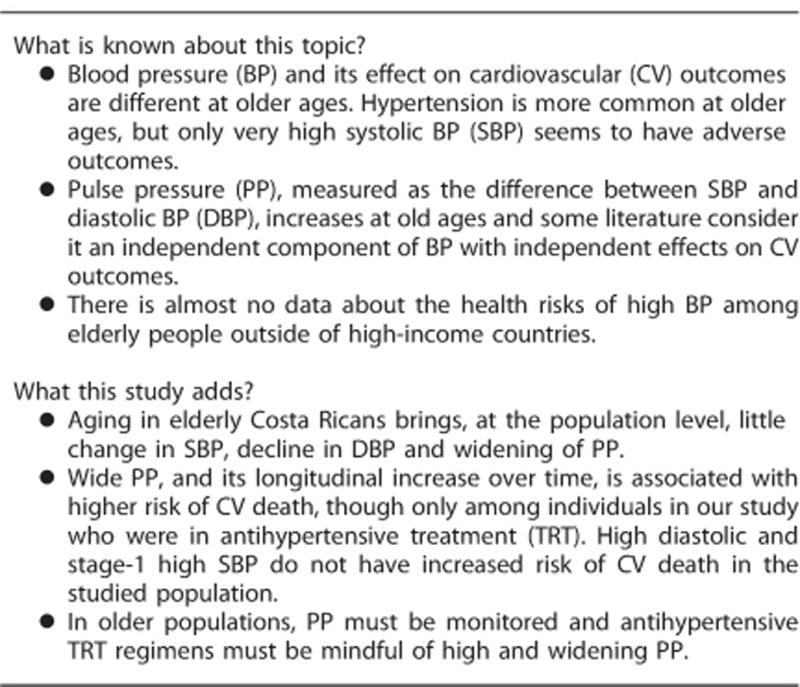


## Figures and Tables

**Table 1:** Characteristics of the study participants by PP group (percentages)

*Characteristics*	*Entire sample*	*PP<70 mm Hg*	*Wide PP*	t*-test of difference*
(*N* at baseline)	(2827)	(1908)	(883)	
				
*Potential confounders*				
Aged 75+	32.2	27.4	43.1	9.46**
Male	47.3	49.0	43.3	−2.89**
Lives in Metro San Jose	51.0	50.8	51.5	−1.44
Completed primary school	49.0	52.7	40.6	−6.52**
Good/ excellent SA economic status	45.4	45.4	45.6	−0.16
Poor/fair SA health	44.3	43.7	45.7	1.42
Current smoker	8.9	8.6	9.7	1.05
BMI<18.5 Kg m^−^^2^	3.0	3.2	2.6	−0.32
BMI⩾30 Kg m^−^^2^	23.0	22.8	23.2	0.25
				
*BP indicators*				
In TRT	47.8	42.3	60.2	9.9**
Stage 1 high SBP 140–159 mm Hg	31.3	30.2	33.8	2.4*
Stage 2 high SBP 160+ mm Hg	21.7	4.1	61.6	42.57**
Low DBP <70 mm Hg	17.6	19.2	14.0	−4.57**
High DBP 90+ mm Hg	22.0	18.2	30.6	8.43**
Wide PP 70+ mm Hg	30.5	0.0	100.0	
				
*BP changes between visits*				
Declined SBP	29.7	22.3	50.3	15.56**
Increased SBP	30.0	34.4	17.3	−10.48**
Declined DBP	29.5	26.3	38.4	6.48**
Increased DBP	12.6	13.1	11.0	−1.74
Declined PP	18.1	11.1	37.7	15.15**
Increased PP	32.6	37.1	19.8	−10.37**
TRT to no-TRT	4.0	3.4	5.3	1.71
No-TRT to TRT	9.9	8.5	13.4	4.11**

Abbreviations: BMI, body mass index; BP, blood pressure; DBP, diastolic BP; PP, pulse BP; SA, self assessed; SBP, systolic BP; TRT, receiving antihypertensive treatment. Declined/increased if change between vists (wave 2 vs 1 and wave 3 vs 2) was ⩾|5| mm Hg per year. Proportion computed using sampling weights and the information of the three waves. Significance: *P<|0.05|, **P<|0.01|.

**Table 2:** Crude CV death rate (per 1000) by BP and TRT classes

*BP*	*Entire sample*			*Not in TRT*		*In TRT*	
	N	*Rate*	*(95% CI)*	*Rate*	*(95% CI)*	*Rate*	*(95% CI)*
*N*				7269		7286	
Total	14 554	13	(12–15)	7	(6–9)	20	(17–24)
							
*SBP*							
No high SBP <140 mm Hg	6327	11	(9–13)	6	(5–9)	18	(14–23)
S1 high SBP 140–159	4576	12	(9–15)	8	(5–12)	15	(11–21)
S2 high SBP 160+	3441	20	(15–26)	8	(5–14)	27	(21–37)
							
*DBP*							
Low DBP <70 mm Hg	3121	19	(15–25)	8	(5–13)	32	(25–43)
Normal DBP 70–89	8461	12	(10–14)	7	(5–10)	17	(14–22)
High DBP 90+	2762	11	(8–16)	6	(3–13)	15	(10–24)
							
*PP*							
PP <70 mm Hg	9028	10	(8–12)	6	(5–8)	14	(12–18)
Wide PP 70+	5313	20	(17–25)	10	(7–15)	27	(22–35)

Abbreviations: BP, blood pressure; CV, cardiovascular; CI, confidence interval; DBP, diastolic BP; PP, pulse BP; SBP, systolic BP; TRT, treatment. N, 2827 subjects (2801with BP information), 14 554 years, 357 CV deaths rates corrected for sampling weights.

**Table 3:** CV death HR of BP levels and TRT. Gompertz regression models

*BP and TRT*	*Traditional model*		*SBP–PP model*		*DBP–PP model*	
	*HR*	*(95% CI)*	*HR*	*(95% CI)*	*HR*	*(95% CI)*
						
*SBP*						
Normal <140 mm Hg	1	Ref.	1	Ref.		
Stage 1 high 140–159	1.02	(0.70–1.50)	0.81	(0.53–1.23)		
Stage 2 high 160+	1.89**	(1.20–2.98)	1.13	(0.70–1.82)		
						
*DBP*						
Low DBP <70 mm Hg	1.25	(0.86–1.81)			1.10	(0.79–1.54)
Normal DBP 70–89	1	Ref.			1	Ref.
High DBP 90+	0.61+	(0.36–1.03)			0.78	(0.49–1.23)
In TRT	2.04**	(1.49–2.78)				
						
*PP and TRT*						
Normal PP<70, no TRT			1	Ref.	1	Ref.
Wide PP⩾70, no TRT			1.00	(0.53–1.89)	1.08	(0.64–1.83)
Normal PP<70, in TRT			1.69*	(1.10–2.60)	1.69*	(1.10–2.60)
Wide PP>70, in TRT			2.58**	(1.53–4.35)	2.82**	(1.83–4.34)
Ratio wide PP/normal PP if in TRT			1.53+	(0.97–2.40)	1.66*	(1.13–2.45)

Abbreviations: BP, blood pressure; CV, cardiovascular; CI, confidence interval; DBP, diastolic BP; HR, hazard ratios; PP, pulse BP; SBP, systolic BP; TRT, treatment. Controlled for age, sex, region, smoking, BMI classes, education and self-assessed health and economic situation. N, 2360 subjects, 12 368 years, 267 CV deaths. Sampling weights included. Significance: *P<|0.05|, **P<|0.01|, +*P*<|0.10|.

**Table 4:** CV death HR of levels and changes in BP by TRT status. Gompertz regression models

*BP levels and changes*	*No TRT* N=*1157*		*TRT*^a^ N=*840*	
	*HR*	*(95% CI)*	*HR*	*(95% CI)*
*SBP and PP model*				
*Initial level*				
Stage 2 high SBP	2.22+	(0.89–5.51)	0.89	(0.50–1.57)
Wide PP	0.94	(0.34–2.58)	2.59**	(1.46–4.58)
				
*Change between visits*				
*SBP*				
Decreased	1.41	(0.62–3.17)	1.50	(0.76–2.97)
Increased	0.93	(0.41–2.11)	0.93	(0.47–1.84)
				
*PP*				
Decreased	0.80	(0.32–2.02)	0.86	(0.46–1.61)
Increased	2.59*	(1.23–5.48)	2.09*	(1.09–3.99)
				
*TRT*				
Started	1.96+	(0.98–3.90)	NA	
Stopped	NA		1.10	(0.56–2.16)
				
*DBP and PP model*				
*Initial levels*				
Low (<70) DBP	1.05	(0.50–2.20)	1.43	(0.85–2.42)
Wide PP	1.57	(0.77–3.22)	2.51**	(1.53–4.14)
				
*Change between visits*				
*DBP*				
Decreased	1.49	(0.76–2.92)	1.19	(0.71–1.99)
Increased	0.97	(0.27–3.50)	0.80	(0.43–1.49)
				
*PP*				
Decreased	0.96	(0.39–2.38)	1.01	(0.56–1.84)
Increased	2.20*	(1.08–4.49)	1.76*	(1.05–2.96)
				
*TRT*				
Started	2.07*	(1.02–4.20)	NA	
Stopped	NA		1.11	(0.56–2.19)

Abbreviations: BP, blood pressure; CV, cardiovascular; CI, confidence interval; DBP, diastolic BP; HR, hazard ratios; NA, not appliable; PP, pulse BP; SBP, systolic BP; TRT, treatment. Controlled for age, sex, region, smoking, BMI classes, education, and self assessed health & economic situation. Sampling weights included. Significance: *P<|0.05|, **P<|0.01|, +*P*<|0.10|. 'Decreased' and 'increased' are changes ⩾|5 mm Hg| per year.

a In TRT if taking hypertension medicines at the begining of the two-visit interval. People in TRT had a HR=2.59 compared with those no in TRT.
