# Supplementary material for: Pulse blood pressure and cardiovascular mortality in a population-based cohort of elderly Costa Ricans
Source: J Hum Hypertens. 2015 Dec 17;30(9):555–62. doi: 10.1038/jhh.2015.117 (PMC4912461; doi:10.1038/jhh.2015.117)
Supplement: Supplementary Information [file jhh2015117x1.pdf]

Table SI-2. Non-CV death Hazard Ratios (HR) of blood pressure levels and treatment. Gompertz regression models

Blood pressure (BP)	Traditional model		SBP-control model		DBP-control model	
and treatment (TRT)	HR	(95% C.I.)	HR	(95% C.I.)	HR	(95% C.I.)
Systolic BP						
Normal <140 mmHg	1	Ref.	1	Ref.		
Stage 1 high 140-159	0.75+	(0.53 - 1.04)	0.70+	(0.47 - 1.03)		
Stage 2 high 160+	0.91	(0.62 - 1.33)	0.81	(0.53 - 1.23)		
Diastolic BP						
Low DBP <70 mmHg	1.22	(0.90 - 1.68)			1.31+	(0.97 - 1.77)
Normal DBP 70-89	1	Ref.			1	Ref.
High DBP 90+	0.97	(0.63 - 1.49)			0.96	(0.65 - 1.42)
In treatment (TRT)	1.15	(0.88 - 1.50)				
Pulse pressure & TRT						
Normal PP<70, no TRT			1	Ref.	1	Ref.
Wide PP>=70, no TRT			0.89	(0.57 - 1.39)	0.78	(0.52 - 1.15)
Normal PP<70, in TRT			1.07	(0.76 - 1.50)	1.05	(0.74 - 1.47)
Wide PP>70, in TRT			1.22	(0.79 - 1.87)	1.07	(0.75 - 1.51)
Ratio wide PP/normal PP if in TRT			1.14	(0.74 - 1.75)	1.02	(0.71 - 1.46)

Controlled for age, sex, region, smoking, BMI classes, education, and self assessed health & economic situation.
N: 2 360 subjects, 12 365 years, 403 non-CV deaths
Excluded 438 subjects with history of heart disease, stroke or cancer at baseline
Sampling weights included.
Significance: ** P<|0.01|, * P<|0.05|, + P<|0.10|
